# Bio-inspired multimodal learning with organic neuromorphic electronics for behavioral conditioning in robotics

**DOI:** 10.1038/s41467-024-48881-2

**Published:** 2024-06-04

**Authors:** Imke Krauhausen, Sophie Griggs, Iain McCulloch, Jaap M. J. den Toonder, Paschalis Gkoupidenis, Yoeri van de Burgt

**Affiliations:** 1 Institute for Complex Molecular Systems, Eindhoven University of Technology, Eindhoven, The Netherlands; 2 Microsystems, Department of Mechanical Engineering, Eindhoven University of Technology, Eindhoven, The Netherlands; 3 Max Planck Institute for Polymer Research, Mainz, Germany; 4 Department of Chemistry, University of Oxford, Oxford, UK

**Keywords:** Electronic devices, Polymers, Information storage

## Abstract

Biological systems interact directly with the environment and learn by receiving multimodal feedback via sensory stimuli that shape the formation of internal neuronal representations. Drawing inspiration from biological concepts such as exploration and sensory processing that eventually lead to behavioral conditioning, we present a robotic system handling objects through multimodal learning. A small-scale organic neuromorphic circuit locally integrates and adaptively processes multimodal sensory stimuli, enabling the robot to interact intelligently with its surroundings. The real-time handling of sensory stimuli via low-voltage organic neuromorphic devices with synaptic functionality forms multimodal associative connections that lead to behavioral conditioning, and thus the robot learns to avoid potentially dangerous objects. This work demonstrates that adaptive neuro-inspired circuitry with multifunctional organic materials, can accommodate locally efficient bio-inspired learning for advancing intelligent robotics.

## Abstract (web summary)

Krauhausen et al. developed a robotic system that learned how to avoid dangerous objects. This behavioural conditioning was demonstrated by forming multimodal associative connections of various sensors, using an integrated organic neuromorphic circuit.

## Introduction

Advancements in the field of robotics have witnessed a notable shift towards bio-inspiration, motivated by the remarkable capabilities of biological nervous systems^1–3^. Bio-inspired robotics introduces novel ways for robots to interact with and be integrated into the physical world. Achieving this goal often necessitates the use of functional materials chosen for their ability to provide the desired flexibility, deformability, or adaptability^4,5^.

At the same time, artificial intelligence (AI) is already demonstrating its proficiency for highly complex tasks in various domains such as data analysis, decision making and computer vision^6^. AI systems mostly utilize large-scale (deep) neural networks for learning, pattern recognition, classification, and language processing inside a static environment^7,8^. These systems are based on gradient-based algorithms that require high computing power and memory storage as well as a large amount of labeled training data. Although these systems are highly effective, their biological plausibility is limited^9^, and they can be power-hungry^10^. Hence, there is a desire to explore alternative bio-inspired algorithms, such as spiking neural networks, genetic and evolutionary algorithms, and swarm strategies, and to further enhance the development of specialized neuromorphic hardware platforms^11–13^. Such innovations in algorithms and hardware have proven to be powerful tools for simulating neural processes, accelerating the training of artificial neural networks, and leading to increasingly sophisticated hardware for artificial neural systems. However, essential adaptive neuronal processes, including associative learning and behavioral conditioning, exist in primitive organisms like the box jellyfish which even lack centralized nervous systems^14^. This raises the question of whether complexity in algorithms and architectures is always imperative for achieving cognitive functions and intelligent behavior. The relatively simple neural circuits of primitive species still exhibit significant capabilities, suggesting that emulating fundamental biological learning principles locally with functional materials and devices could be equally important as complexity while gaining efficiency^4,15^.

Primitive biological organisms employ fundamental strategies for learning, such as exploration, multimodal processing, and behavioral conditioning. From early developmental stages, living beings instinctively start to learn from experience and through trial and error by interacting with their surroundings^16^. During this initial exploration phase, behaviors tend to be somewhat random and lack a specific goal while the organism is engaged with the environment via a wide range of sensory modalities (touch, vision, olfaction, etc.). The randomness of certain behaviors, such as bumping into an object, leads to the discovery of new sensations and, consequently, learning opportunities. Through this physical interaction of organisms with their surroundings, behavioral randomness develops gradually into consistency^17^. In this context, multimodal sensing enables the collection of various sensations describing the same event. These concurrent multimodal observations are synchronized in time and, as a result, become correlated, establishing autonomic connections across different sensory modalities and enabling behaviors such as respondent (Pavlovian) conditioning and associative learning. Indeed, a recent study of the complete connectome of a Drosophila brain reveals that the majority of neurons process multimodal signals^18^. Adaptivity and plasticity in function and behavior - essentials for biological development - are especially effective if previous experiences and memory are taken into account as well^19^. For instance, behaviors are associated with consequences through affirmative (rewards or reinforcement) and adverse (punishment) stimuli to strengthen or weaken a specific behavior (operant conditioning). By providing diverse sensory feedback and abundant opportunities to learn from the environment, explorative behavior and multimodal processing allow for instruction-free processes that converge into optimal behavioral conditions via adaptivity.

Emerging functional materials and devices can offer unique properties that go beyond what conventional systems and electronics could achieve^20^. Organic mixed ionic-electronic (semi)conductors have recently experienced a notable upswing in neuromorphic engineering^21–23^. They are able to replicate bio-inspired functionalities such as synaptic plasticity^24,25^, neural processing^26^, high connectivity and recurrence^27,28^ and even forgetfulness^29^ just by material-inherent mechanisms. Key features of organic synaptic devices are their adaptivity through linear, symmetric, and analog tuning of electrical conductance and their operation at low voltage with high energy efficiency^30^. The compatibility of organics with solution-based processes and large-area integration into flexible or stretchable substrates can enable the merging of organic neuromorphic electronics in unconventional form factors (body, robotics, buildings, etc.)^31^. Indeed, significant steps have been made using conductive polymers regarding localized handling of data via on-chip training^32,33^, real-time operation with online learning^34^ and spiking circuits for bio-integration^35,36^. Despite these significant demonstrations, applications are often limited to abstract and conceptual demonstrations in well-defined laboratory settings or mock environments, enabled by simple binary decisions. Robotic setups offer a realistic platform for interaction-rich, real-life setups^37^. Robotic manipulators, for example, are crucial for a variety of applications serving in versatile and dynamic environments, ranging from industrial assembly lines to neural prostheses. Highly adaptive and localized control close to the sensory nodes can drastically improve performance and can also warrant operational safety which is essential for human-oriented purposes such as neuroprosthetics^38,39^.

In this work, we present a robotic system that uses multimodal sensory stimuli to explore and interact with a real-world environment in real time while adapting to it using bio-inspired mechanisms. At the core of adaptivity and learning of the robotic system is an organic neuromorphic circuit that consists of organic electrochemical transistors (OECTs) and organic neuromorphic devices (also called electrochemical random-access memories, ECRAMs). This bio-inspired approach enables the robotic agent to incrementally learn and perform a complex behavioral task, showcasing its adaptability and distributed intelligence in responding locally to dynamic and multimodal environmental cues. More specifically, the robotic system gains the ability to distinguish between safe and potentially harmful objects through local adaptation of neuromorphic circuitry. This work demonstrates that highly functional organic materials can reform neuromorphic hardware, rethinking adaptive intelligent systems as small(er)-scale local circuitry that interacts with the environment with bio-inspired learning mechanisms.

## Results

**Fig. 1:**
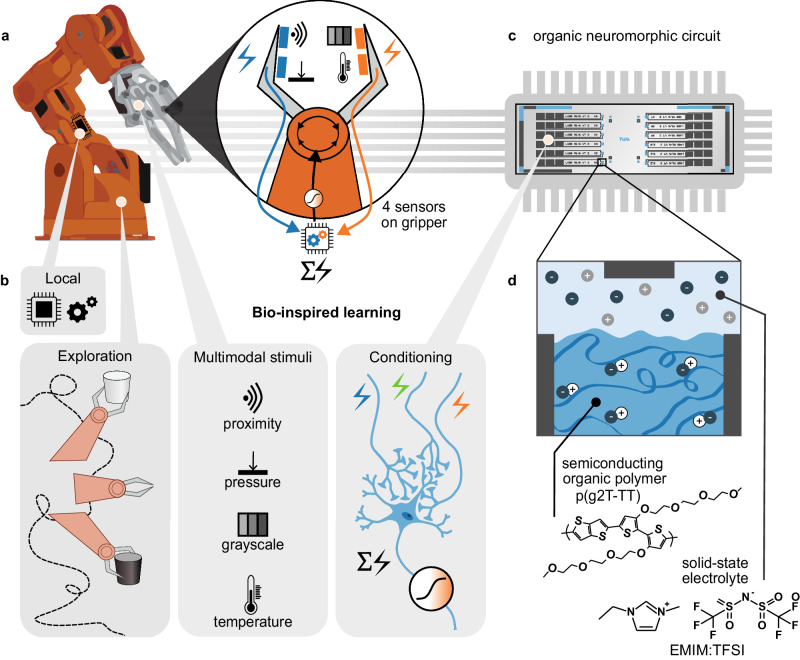
Robotic manipulator with an organic neuromorphic circuit using bio-inspired learning. **a** A robotic manipulator with a custom-made gripper is equipped with four multimodal sensors. The sensory stimuli are processed adaptively via specialized hardware and condition the grasp behavior of the robotic system. **b** The robot employs the following bio-inspired principles for learning: an exploration of its environment through random movement, collection of multimodal sensory inputs and adaptive processing leading to behavioral conditioning. **c** The robotic system is connected to a local organic neuromorphic circuit that emulates neuronal processing, such as short-term and long-term synaptic plasticity and dendritic summation. The neuromorphic circuit consists of organic electrochemical devices. **d** Schematic architecture of an organic electrochemical device based on the semiconducting polymer p(g2T-TT) and a solid-state electrolyte based on the ionic liquid EMIM:TFSI. The device is defined by three electrodes (gray): source (left), drain (right) and gate (top). The polymer is distributed between the source and drain terminals (blue) and exhibits mixed electronic-ionic conduction. Anions (dark blue) from the electrolyte can penetrate into the polymer bulk leading to the formation of holes (white) along the polymer backbone and changing its conductivity. The drawing of the full robotic arm in Fig. 1a is based on the Arduino® Braccio Kit image by Arduino under the CC BY-SA license.

The robotic system is based on the Arduino Braccio Kit (Fig. 1a), with five degrees of freedom and an additional movement option for opening and closing a gripper. The gripper acts as the hand of the robotic manipulator and is equipped with four sensors that continuously collect multimodal sensory stimuli of pressure, distance, temperature, and color tone when manipulating objects (Figs. 1a and 1b). A custom gripper setup is realized to accommodate the collection of multimodal sensory signals in a hand-like shape (Fig. S1 and “Methods” section). Different cups (dark/hot, white/cold) are placed sequentially near the robotic system so that it is able to either pick them up or refuse them. Each movement of the robot follows an autonomic sequence of specified moves that provides a behavioral baseline for any action taken. The movements vary between a pick-up action with a grab or no-grab option in the end, a drop action that concludes a successful grab, and a pull-back action to avoid the cup that functions as a no-grab. These actions are driven via an Arduino Uno that operates the motors of the robotic setup. The motor commands are continuously modulated by sensory stimuli from the environment, i.e., a detection of a cup in close proximity with the hand or a pressure applied due to a successful grab, creating a real-time response of the robot to its surroundings (that is, the object of interest). Without any prior external influence, the robot is in an explorative state in which it incidentally picks a cup or not with the grab or no-grab actions initially taken randomly (Fig. 1b). Whenever a cup is discovered (grabbed) by chance, it inherently leads to new sensory sensations. An analog trainable neuromorphic circuit (Figs. 1a and 1c) interacts locally with the sensory signals and allows learning via adaptive associative connections necessary for behavioral conditioning (Fig. 1b, right). The organic neuromorphic circuit comprises of organic electrochemical devices, OECTs and ECRAMs, that are either volatile or non-volatile respectively (Fig. 1d). The output voltage $\sum V$ of the organic neuromorphic circuit depends on the conductance state of each organic electrochemical device and reflects the sensory signals in an event-driven nature. $\sum V$ merges the input branches of electrical circuitry similar to the dendritic summation of multiple neurons via the synapses (Fig. 1b, right).

**Fig. 2:**
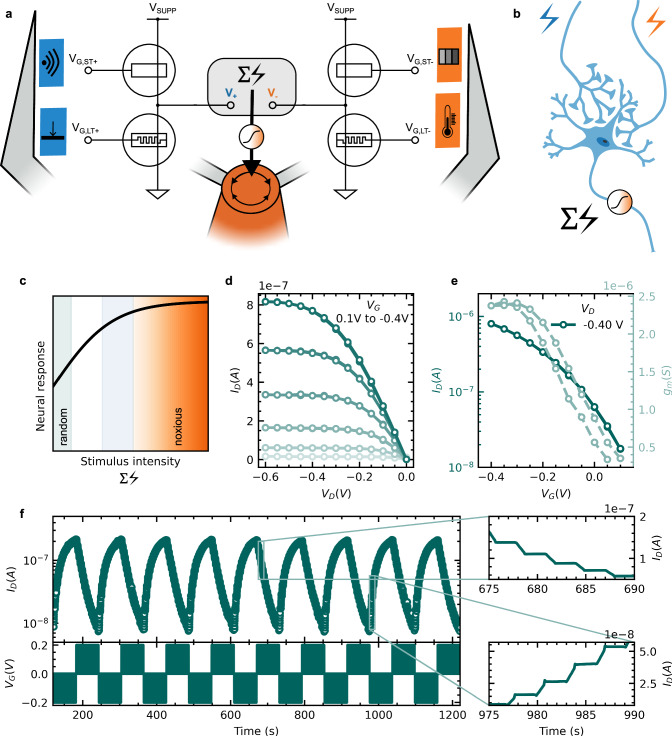
Characterization of the organic neuromorphic circuit. **a** Circuit schematic of the organic neuromorphic circuit following a two-branch (+ and -) architecture. The gate of each organic electrochemical device is connected to a specified sensory stimulus. The sum $\sum V$ over the output voltages ${V}_{+}$ and ${V}_{-}$ branches is forwarded to the robotic system via an activation function. **b** Biological representation of dendritic summation involving two presynapti**c** signals. **c** A sigmoidal activation function translates the stimulus intensity $\sum V$ into a neural response (probability for a certain behavior, from random (green) to noxious (orange)). **d** Output characteristics of the volatile synaptic device that displays short-term memory. **e** Transfer characteristics of the volatile synaptic device that displays short-term memory. **f** Long-term memory of the non-volatile synaptic device.

The organic neuromorphic circuit consists of four micrometer-scale organic electrochemical devices (Fig. S2 and “Methods” section), mimicking synaptic plasticity and, therefore, exhibiting neuro-emulating functionality. Two of these devices function as OECT and operate in a volatile, short-term manner (indicated as ST). The other two devices operate in a non-volatile manner as ECRAM with long-term effects (referenced as LT, Fig. 2a). The four devices are arranged in two branches (+ and -) that each contains a volatile and a non-volatile element in series. The combined output voltage is the sum over both branches: $\sum V={V}_{+}+{V}_{-}$. This closely resembles the dendritic summation of multiple presynaptic signals at the synapses of a postsynaptic neuron (Fig. 2b). Each branch also displays an intrinsic associative adaptation due to the interplay of OECT and ECRAM. If loaded with a (adaptive) resistive load, the OECT changes its operating regime and thus its transconductance (Fig. S3 and S4). The transconductance represents a tunable sensitivity towards the sensory stimuli that can be strengthened or weakened via the ECRAM leading to an inherent association between the two stimuli at OECT and ECRAM.

The output voltage $\sum V$ is translated into a motor action through an activation function that relates the signal to a behavioral probability (Fig. 2c). The activation function is sigmoidal and proportional to the widely used activation function hyperbolic tangent (tanh), converging around 1. It is executed on the Arduino Uno and while this is part of the processing, it only provides a static, fixed translation of an analog output voltage such as $\sum V$ into a behavioral movement pattern. The output voltage is also interpreted in terms of probability, which means only determines the probability for a certain action, but not necessarily the action itself. The non-deterministic and fail-prone behavior in biological systems causing new sensations is one of the reasons for their remarkable adaptability in unknown situations^40^. While the Arduino Uno relays signals from the organic neuromorphic circuit to the robotic setup, it operates solely as a translator/mediator and has no agency on the behavior of the robotic agent. In order to react to the environment, the neuromorphic circuit handles optical, thermal, and mechanical stimuli. A color and proximity sensor are used for gaining information on objects (i.e., a cup) from afar/without contact and drive the gates and thus (trans-)conductance of the volatile devices, ${G}_{{ST}+}$ and ${G}_{{ST}-}$. A pressure and temperature sensor feed a signal on contact to the non-volatile gates of the neuromorphic circuit, ${G}_{{LT}+}$ and ${G}_{{LT}-}$ providing the necessary impulses for learning and conditioning. Via the series connection in the circuit layout, the (+)-branch then combines the sensory input of pressure and proximity in a single information stream leading to the output voltage ${V}_{+}$. This functionality is mirrored in the (-)-branch coupling temperature and color resulting in signal stream ${V}_{-}$. We employ off-the-shelf sensors for collecting sensory input which provides lifelike, noise-containing data (Fig. S5, see sensor section in “Methods”). The sensory signals undergo basic pretreatment through an additional analog hardware unit to align with the low operating voltages (≤1.0 V) of the neuromorphic devices (Fig. S6, see sensor section in “Methods”).

The robotic system follows its movement patterns remaining in an explorative state until it starts interacting with the environment and receives new sensory stimuli. These stimuli change the output voltage $\sum V$ momentarily or permanently leading to an event-driven and adaptive behavior.

The neuromorphic circuit consists of volatile (OECTs) and non-volatile (ECRAMs) organic electrochemical devices. These devices utilize the semiconducting polymer poly(2-(3,3′-bis(2-(2-(2-methoxyethoxy)ethoxy)ethoxy)-[2,2′-bithiophen]−5-yl) thieno [3,2-b] thiophene) [p(g2T-TT)] as the channel material and are controlled through an electrolyte. The modulation of the electronic current within the channel, specifically the conductance state, is achieved through the application of an ionic gate current^41^. The polymer p(g2T-TT) displays mixed ionic-electronic conduction by supporting the transport of both holes and ions. This polymer serves as a versatile platform for various functionalities and is suitable for both short- and long-term devices depending on the probing conditions^34,42^. Hence, the organic neuromorphic circuit allows for monolithic integration of both volatile and non-volatile functionalities with the same polymer as the channel material of the transistors. It exhibits a wide range of well-defined conductance states (with a > 100 on/off ratio), high linearity, sensitivity to gate pulses (ranging from μS to mS), and stability (>10^9 write-read operations)^42,43^. The low-voltage operation (≤ ± 1 V) and compatibility with solution-based processing methods contribute to high energy efficiency and cost-effectiveness. While short-term (volatile) and long-term (non-volatile) synaptic devices share a similar device architecture, their primary distinction lies in the device configuration. For the short-term effect, the gates are directly linked to the sensor signal. Conversely, in non-volatile devices, a switch with a current-limiting resistance of $100M\Omega$ is connected in series to the gate, inducing an open-circuit potential when no sensor signal is applied (see Methods). This induces a lasting change in conductance, inducing long-term (non-volatile) synaptic memory phenomena. We adopt a side-gate device architecture with a solid-state electrolyte comprised of the ionic liquid [1-ethyl-3-methylimidazolium bis(trifluoromethylsulfonyl)imide (EMIM:TFSI) embedded in a polyvinylidene fluoride-co-hexafluoropropylene (PVDF-HFP) polymer matrix (see Methods).

The device characteristics of the neuromorphic circuit are shown in Figs. 2d–2f in the face of the volatile and non-volatile synaptic devices respectively. We attain low voltage operation for all components of the organic neuromorphic circuit and write currents <5 nA and conductance values < 100nS for the ECRAM (Fig. S7) indicating low energy demands of the circuit^30^. We achieve stable performance with a minimal hysteresis for the volatile synaptic device as shown in the output (${I}_{D}$ over ${V}_{D}$) and transfer (${I}_{D}$ over ${V}_{G}$) characteristics (Figs. 2d and 2e, respectively). The transconductance ${g}_{m}$ (Fig. 2e), also described as the device sensitivity, depends on the gate voltage but can also be influenced via the drain voltage. An OECT switched in series with a resistive load ${R}_{L}$ moves its operation from linear to saturation depending on ${R}_{L}$ as detailed in^44^. The ratio of resistances between load and OECT is critical and a substantial ratio change ($\frac{{R}_{{OECT}}}{{R}_{L}}=1\to 50$) is necessary to achieve a significant change in the output voltage (${V}_{{OUT}}=\,\frac{{V}_{{SUPP}}}{2}\,\to 0V$) and in the amplification of the gate voltage through the transconductance (Fig. S3). An additional measurement of the voltage output for an OECT loaded with different resistances is provided in Fig. S4. Replacing the resistive load ${R}_{L}$ with the non-volatile synaptic device (LT), as in our circuit topology, prompts similar changes in voltage level for the branch voltages ${V}_{+}$ and ${V}_{-}$ and in the transconductance of the OECTs. This change in transconductance of the OECT and therefore change in output voltage causes an inherent link between the two gate stimuli, a form of associative learning. Figure 2f shows the programming characteristics of the non-volatile synaptic device. which displays high on-off ratio across orders of magnitude with linear switching behavior and stable state retention (zoom-ins) for long-term plasticity at very low programming voltage ($V\le \left|0.2V\right|$). The conductance states are adjusted reversibly by applying gate pulses of opposite polarity. These long-term conductance changes in the artificial synapses create the memory effect needed for learning and adaptive behavior.

**Fig. 3:**
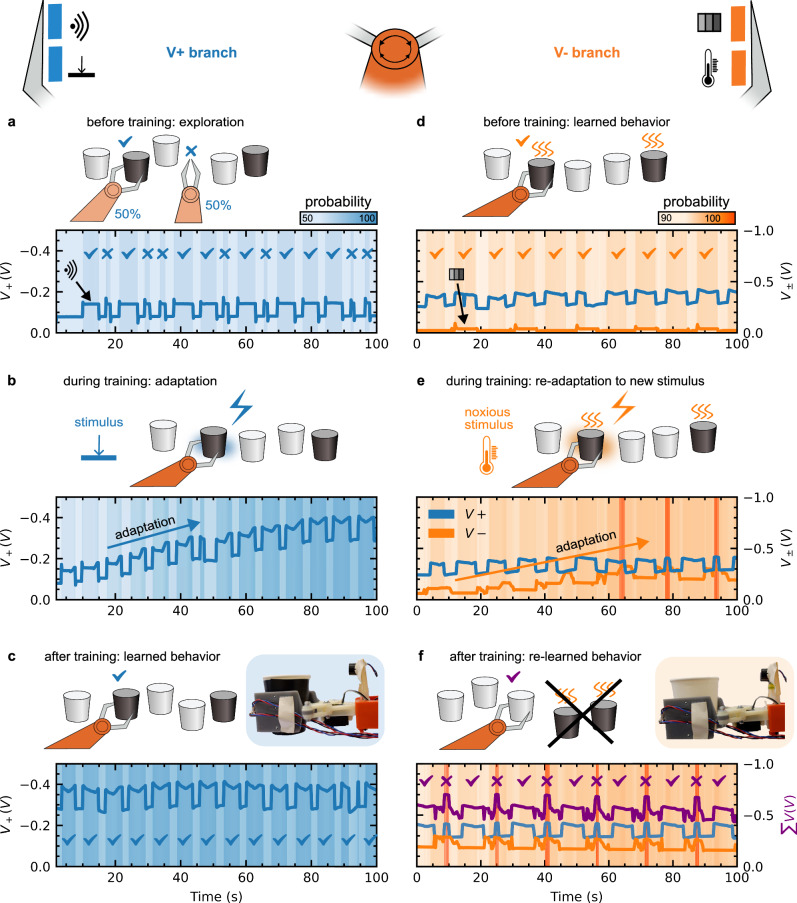
Behavioral change of the robotic manipulator upon adaptive processing of multimodal stimuli. **a** Explorative behavior of the robot before any adaptation. A checkmark (✓) represents a successful grab of the cup, the cross (✗) indicates a missed or refused grab attempt. **b** Adaptation of the ${V}_{+}$ (blue line) branch to the pressure stimuli when incidentally grabbing a **c**up. **c** After training, the robotic manipulator consistently grabs the cup if it is close by, when detected by the proximity sensor. The inset image depicts the robot holding a (dark) cup. **d** Established behavior from the ${V}_{+}$ branch is maintained while the addition of new stimuli via the ${V}_{-}$ (orange line) branch has no effect. **e** Adaptation to the new temperature stimuli in the ${V}_{-}$ branch. **f** After training, the robotic manipulator only grabs cups that are white and cold, but not those that are dark and hot. The final voltage output $\sum V$ is shown as purple line. The inset image pictures the robot holding a white cup.

Overall, the learning process of the robotic manipulator is shown in Fig. 3. The organic neuromorphic circuit combines the collection of multimodal sensory stimuli with neuronal processing leading to associative connections and behavioral consequences. Therefore, the robot learns to avoid potentially harmful objects like a hot cup. Initially, the robotic system is an explorative state in which it experiments with different behaviors, in this case grabbing or non-grabbing action (Fig. 3a). As a baseline behavior, the robotic system is already able to grab a cup, but this occurs at random and is unrelated to any external stimuli (i.e., the trait of a cup). It operates undirected and associative conditioning is latent and thus yet to be formed. Sensory cues are already present but lead to no change in behavior via the activation function. Initially, only standard (cold) cups are used as objects which render the (-)-branch (Figs. 2a and 2b, orange bolt) of the neuromorphic circuit reacting to temperature inactive for now. An object (i.e., a cup) gets registered by the proximity sensor, causing a short-term peak of ${V}_{+}$ and subsequently of $\sum V$ (Fig. 3a). A longer peak in this context means that the cup is picked up (checkmark ✓) and held until the follow-up drop action, a shorter peak indicates that the cup is indeed detected but not grabbed (cross ✗) (Fig. 3a and Movie S1). To showcase the random behavior of the robotic agent over time without learning, the training signals are disconnected from the non-volatile synaptic device for this experiment to prevent any adaptation. With all sensor connections restored, the organic neuromorphic circuit adapts to the sensory cues from its environment. Whenever the robot successfully grabs a cup, the pressure sensor on the gripper directly forwards a signal to the non-volatile synaptic device (${V}_{G,{LT}+}=\pm 0.5V$). This happens in addition to the peak shown before, which was provoked by a pulse from the proximity sensor at the gate of the OECT (${V}_{G,{ST}+}=\,-0.25V$). The activation leads to an increase in voltage ${V}_{+}$ (Fig. 3b). The probability for a grab behavior therefore changes represented as the background color (light to darker blue) in Fig. 3 and consequently the overall behavior shifts from random to systemic (Movie S2). A darker blue tone indicates a high probability of grabbing a cup. From Fig. 3b, it is apparent that a certainty in behavior develops only for the simultaneous occurrence of long-term synaptic change (increase in general voltage level of ${V}_{+}$) and the short-term change during the detection of an object (peak in ${V}_{+}$). In between peaks (that is, in between object detections) the probability declines again (lighter blue), so an inherent associative link between object proximity and the grabbing action (the training pressure signal) is formed, similar to biological associative learning or respondent conditioning (Pavlovian response). Complete adaptation is achieved after 14 training steps and the robotic manipulator consistently grabs the cup if it is close by (Fig. 3c, checkmarks and Movie S3). This behavior is also resistant to instabilities and imperfect sensor signals that can be caused by non-optimal grip and/or shifting and slipping of the object during grasping (seen in the last peak of the measurement, Fig. 3c at 90–95 s) and maintained stably over time and under movement.

Complex tasks can often be broken down into smaller components that are learned separately and incrementally. This technique is called chaining and is well-known in research fields like behavioral psychology and deep learning^45,46^. Chaining involves teaching a series of behaviors in a specific sequence. Each behavior serves as a cue for the next one. After completing the first cycle of learning, a second behavioral change is built on top (chained), concluding in the fulfillment of a more complex task: The robotic system now faces cups of different temperatures (cold and hot), which are mirrored in their color: a cold cup is white, and a hot cup is dark. Introducing this new thermal stimulus, the (-)-branch connected to the related sensor signals (temperature and grayscale/color) is also active. In the initial state, the previously learned behavior is maintained (Fig. 3d and Movie S4). The (+)-branch (${V}_{+}$ in blue) follows the adapted behavior from before. The (-)-branch yields a small voltage ${V}_{-}$ (in orange) and a peak reaction to the color of the dark (hot) cups. The probability output of the activation function is depicted as an orange hue in the background. Cold and hot cups are handed alternately. Initially, the robot again grabs the cup every time it comes close, disregarding the temperature or color (Fig. 3d, checkmarks) as it has learned to do previously. However, the new thermal stimulus induces a gate voltage at the second non-volatile device (${V}_{G,{LT}}=\,\pm 0.5V$), causing a change in voltage level ${V}_{-}$ and increasing the response in output voltage (peak height) towards a color stimulus. Like in the first training process, an association between the temperature and color is formed, resulting in an associative link (Fig. 3b and Movie S5). Color is thus coupled to temperature. After 4 training steps, the activation function with $\sum V$ as input reaches a very high stimulus intensity (Fig. 2c, probability >100%) forcing a protective reaction of the robotic hand. It draws back and avoids the object. This overstimulation – noxious behavior – only occurs when a hot (and dark) cup is detected, highlighted in Figs. 3e and 3f in dark orange. This progresses our initial adaptation from respondent/Pavlovian learning to a more complex behavior of operant conditioning by learning from positive (pressure) and negative (temperature) consequences of different stimuli. At the end of the whole training process, by including both branches (${V}_{+}$, ${V}_{-}$ and $\sum V$), the robotic system is able to distinguish between two types of cups, essentially classifying dangerous and non-dangerous objects. More specifically, by following and adapting to the dynamic cues of the environment, the robot learns to avoid potentially harmful objects like a hot cup while actively engaging with other safe objects. Figure 3f and Movie S6 present the final output signals and behavior. Both color and temperature sensors are more sensitive to positioning (seen as noisy signals in the measurements), demonstrating a high tolerance for stimulus variations in the learning scheme.

## Discussion

Taking inspiration from the versatile capabilities of biological systems, we combine bio-inspired processing, learning, and control paradigms with the development of organic neuromorphic circuitry, and we demonstrate a standalone robotic system that interacts intelligently with a non-static environment. Through the integration of an organic neuromorphic circuit, the system adapts its behavior based on multimodal sensory feedback from environmental cues. The synaptic devices in the circuit enable associative learning, leading to both respondent (Pavlovian) conditioning and more complex operant conditioning. The robotic agent learns to associate positive and negative consequences with multimodal stimuli, showcasing adaptability and the ability to distinguish between safe and potentially harmful objects. The use of functional materials, such as organic (semi-)conducting polymers, in the neuromorphic circuit is elemental to the system’s capabilities, replicating bio-inspired functionalities like synaptic plasticity, dendritic summation, and neural processing. This is possible using small-scale, locally integrated, and low-voltage monolithic polymer electronics. Moreover, due to the modular-like structure of the neuromorphic circuit, the concept can be extended into multiple branches in order to handle sensory signals of arbitrary complexity and multimodality. The presented robotic system serves as a tangible example of how combining bio-inspired principles with localized organic neuromorphic circuitry can lead to the development of highly adaptive, intelligent, and efficient systems for real-world applications.

## Methods

### Device fabrication

Standard microscope glass slides (75 mm by 25 mm) are cleaned in a sonicated bath, first in a soap solution (Micro-90) and then in a 1:1 (v/v) solvent mixture of acetone and isopropanol. Gold electrodes for the source, drain, and gates are photolithographically patterned [with negative photoresist AZ nLof2035 (MicroChemicals) and AZ 726MIF Developer (MicroChemicals)] on the cleaned glass slides. A chromium layer is deposited to achieve better adhesion of the gold. The photolithography foil masks are designed using KLayout^47^ and the complementary pypi-package koala^48^. Each glass slide contains twelve devices with fixed dimensions. The channel dimensions of the non-volatile devices (LT) are as follows: W/ L = 1/3 with L = 250 μm with a lateral gate of the 1000 μm by 1000 μm and 150-μm distance between the gate and the channel. The volatile device (ST) has the following dimensions: W/L = 1/6 with L = 500 μm with a lateral gate of 1000 μm by 1000 μm and 150-μm distance between the gate and the channel. The complete layouts are depicted in Fig. S2. Two layers of parylene C (Specialty Coating Systems) are deposited. Soap solution (Micro-90 soap solution, 2% (v/v) in deionized water) is used for separation between the layers, allowing the peel-off of the upper layer. An adhesion promoter (silane A-174, Specialty Coating Systems) is added to the lower layer of parylene C to prevent detachment. This layer insulates the gold electrodes. In a second photolithography step using positive photoresist AZ 10XT (MicroChemicals) and AZ Developer (MicroChemicals), the channel and lateral gate dimensions of the devices are defined. Reactive ion etching with O2 plasma is used to carve out the channel and corresponding gates. The semiconducting polymer p(g2T-TT) is synthesized according to (41) and prepared and applied following the procedure in^42,43^. p(g2T-TT) is solved in chloroform (3 mg/ml) inside an N2-filled glove box and spin-cast inside the N2-filled glove box at 1000 rpm for 1 min. The devices are baked at 60 °C for 1 min.

In ambient, the sacrificial upper parylene C is peeled off to confine the polymer inside the gate and channel regions. Excess soap is rinsed off with de-ionized water. An ionic gel is prepared as electrolyte according to^49^. An ionic liquid 1-Ethyl-3-methylimidazoliumbis(trifluoromethylsulfonyl)imide (EMIM:TFSI, Merck) and the copolymer poly(vinylidene fluoride)‐co‐hexafluoropropylene (PVDF-HFP) are solved in acetone inside an N2-filled glove box in the following proportions: 17.6 weight% (wt%) ionic liquid, 4.4 wt% copolymer, and 78 wt% acetone. The solution is stirred for at least 2 hours at 40 °C inside the glove box. The ionic gel is drop-cast with a pipette onto each channel and gate under ambient conditions and dried overnight (Fig. S2).

### Measurements

For measurements of the electrical characteristics of volatile and non-volatile devices, a Keithley 2602B SourceMeter is used. The measurements of the volatile device (ST), the source measure units at the three device terminals are directly connected with the measurement system. For non-volatile measurements (LT), a mechanical switch in series with a resistance ${R}_{G}=100M\Omega$ is added between the gate of the device and the measurement system and enhances the analog memory phenomena. The switch forces open-circuit potential conditions between the gate and channel, while the gate resistor ${R}_{G}$ downscales and limits the gate current in the range of nanoamperes. The probing conditions determine whether the device operates in a volatile or non-volatile manner. With the addition of a switch and current limiting resistance, the charges are effectively trapped inside the polymer channel leading to a non-volatile device behavior^25^. Conversely, without the additional components at the gate, charges inside the semiconducting polymer are not hindered by a high energy barrier and can move around, and thus the behavior is volatile.

### Sensors

The robotic sensors are off-the-shelf components, operate in the analog domain, and are Arduino-compatible. The proximity (URM09 ultrasonic distance), grayscale and temperature (LM35 temperature) sensor are from the DFRobot Gravity line. The pressure sensor uses the Grove force sensor module with a rectangular Taiwan Alpha force sensor pad (MF02-N-221-A01). The sensors and detailed specifications are depicted in Fig. S5. The sensor signals are pretreated with additional analog circuitry shown in Fig. S6. This pretreatment consists of downscaling the sensor output voltage (≤1.0 V) and fixing the output polarity (+/-) to a level suitable for organic devices. The additional circuitry also adds an activation threshold for each sensor, meaning the sensors only forward a signal once it surpasses a certain intensity. The pretreatment is fixed throughout all experiments.

### 3D-printed parts

The custom robotic gripper is designed using Autodesk Inventor and is then 3D-printed using a Formlabs SLA resin printer, model 3. For the gripper, Tough1500 resin is used to allow for slight flexibility and bend. To attach the ultrasonic sensor in front of the gripper, clear resin is used for the printed fixture. All fixtures are shown in Fig. S1 and S5.

### Supplementary information

Supplementary Information

Movie S1

Movie S2

Movie S3

Movie S4

Movie S5

Movie S6

## Data Availability

The data generated in this study are provided in the Supplementary Information and Source Data files.

## Code availability

Code and additional software (robotic control, measurement control, mask design) are available upon request from the authors. Arduino® is a trademark of Arduino SA.
