# Supplementary material for: Bio-inspired multimodal learning with organic neuromorphic electronics for behavioral conditioning in robotics
Source: Nat Commun. 2024 Jun 4;15:4765. doi: 10.1038/s41467-024-48881-2 (PMC11150455; doi:10.1038/s41467-024-48881-2)
Supplement: Supplementary file 1 — Supplementary Information [file 41467_2024_48881_MOESM1_ESM.pdf]

Supplementary Materials

for

# Bio-inspired multimodal learning with organic neuromorphic electronics for behavioral conditioning in robotics

Imke Krauhausen,[1,2,3] Sophie Griggs,[4] Iain McCulloch,[4] Jaap M. J. den Toonder,[1,2] Paschalis Gkoupidenis,[3]* Yoeri van de Burgt[1,2]*

[1]Institute for Complex Molecular Systems, Microsystems, Eindhoven University of Technology, The Netherlands.

[2]Institute for Complex Molecular Systems, Microsystems, Eindhoven University of Technology, The Netherlands.

[3]Max Planck Institute for Polymer Research, Mainz, Germany.

[4]Department of Chemistry, University of Oxford, United Kingdom.

**This PDF file includes:**

- Supplementary Figures S1-S7
- References

**Additional supplementary materials for this manuscript include:**

- Supplementary Movies S1-S6

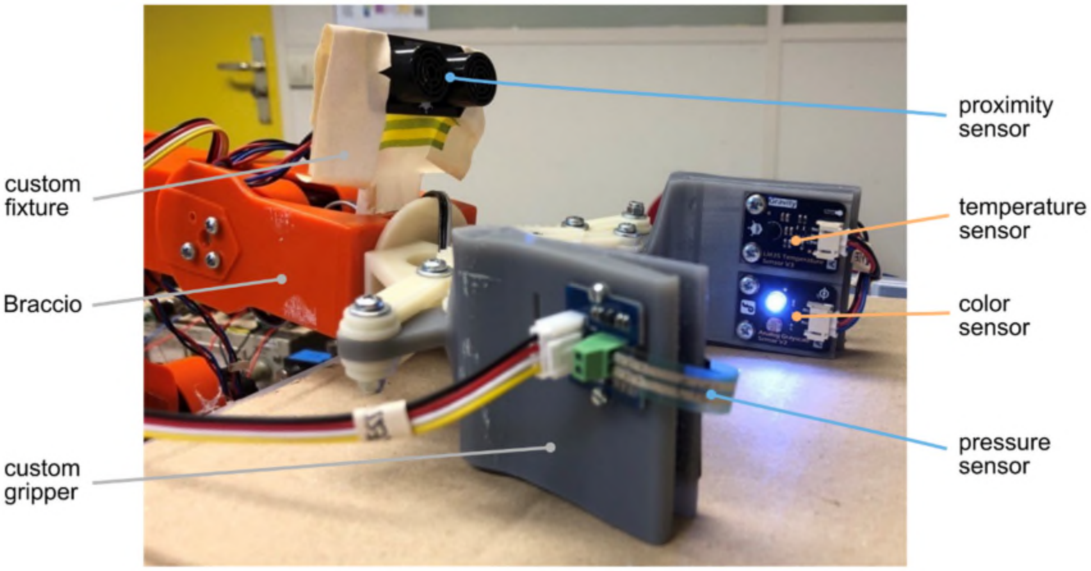


**Fig. S1. Robotic setup.** The robotic arm (orange) is built from the TinkerKit Braccio by Arduino. It has a custom-made 3D-printed hand to accommodate all sensors. The robotic arm is controlled via the Braccio Arduino shield on top of an Arduino Uno. A second Arduino Uno is used to collect the data from the sensors and the neuromorphic electronics via bipolar analog-digital converters as the Arduino itself is limited to reading positive voltages.

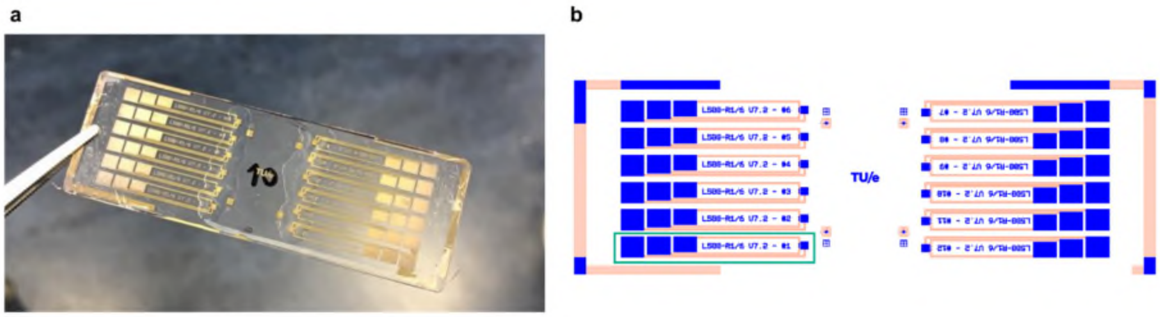


**Fig. S2. Layout of the organic neuromorphic device.** (**a**) Photograph of an exemplary glass slide with twelve organic neuromorphic devices. The ionic gel is visible on top of the gate and channel area of the devices. (**b**) Exemplary mask layout (W/L=1/6, L=500µm) for the organic neuromorphic device (blue=polymer, orange=gold). The glass slide contains twelve devices, one device is marked with a green box.

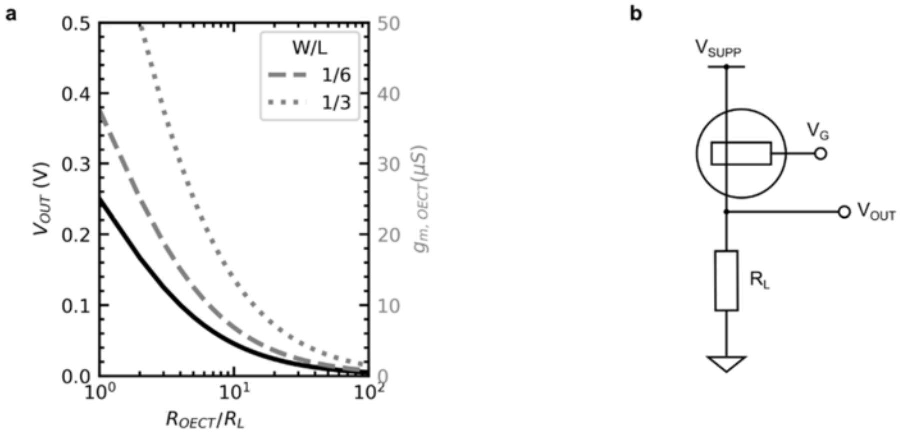


**Fig. S3. Volatile characteristics for the organic electrochemical transistor with different resistance loads.** (**a**) Dependence of the OECT signals on the ratio between OECT resistance and a load resistance. (**b**) Exemplary circuit of an OECT with a load resistance.

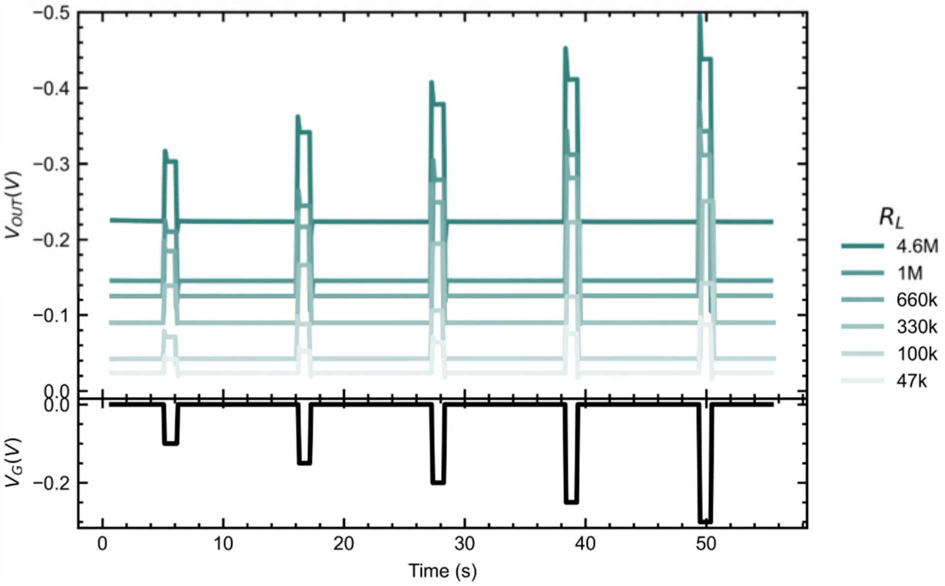


**Fig. S4**. Measurement of the output voltage of an OECT with different load resistances.

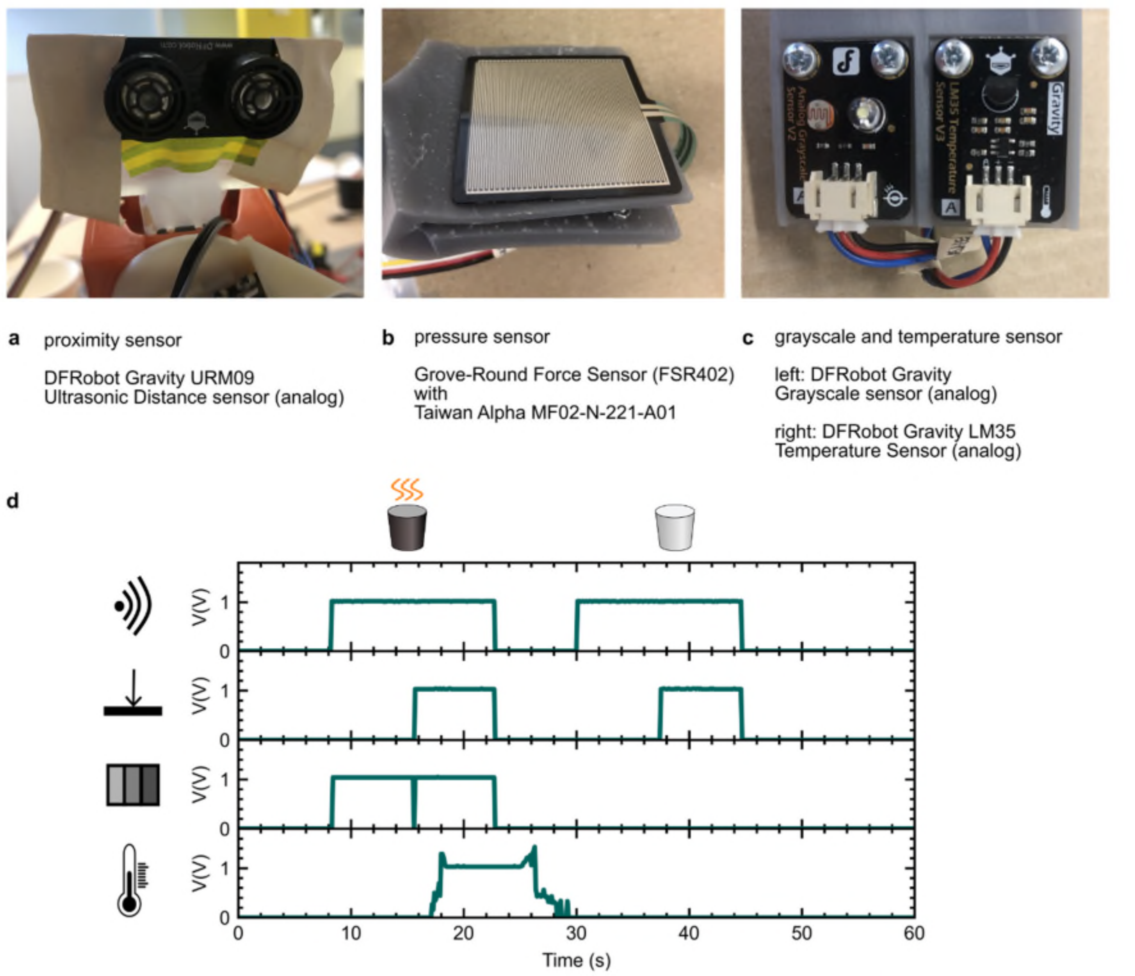


**Fig. S5. Sensor setup.** (**a**) Proximity sensor attached to custom fixture. (**b**) Pressure sensor attached to right custom gripper. (**c**) Grayscale and temperature sensor attached to left custom gripper. (**d**) Exemplary measurement of the sensor signals with +1V sensor output voltage for a hot, dark cup and a cold, white cup.

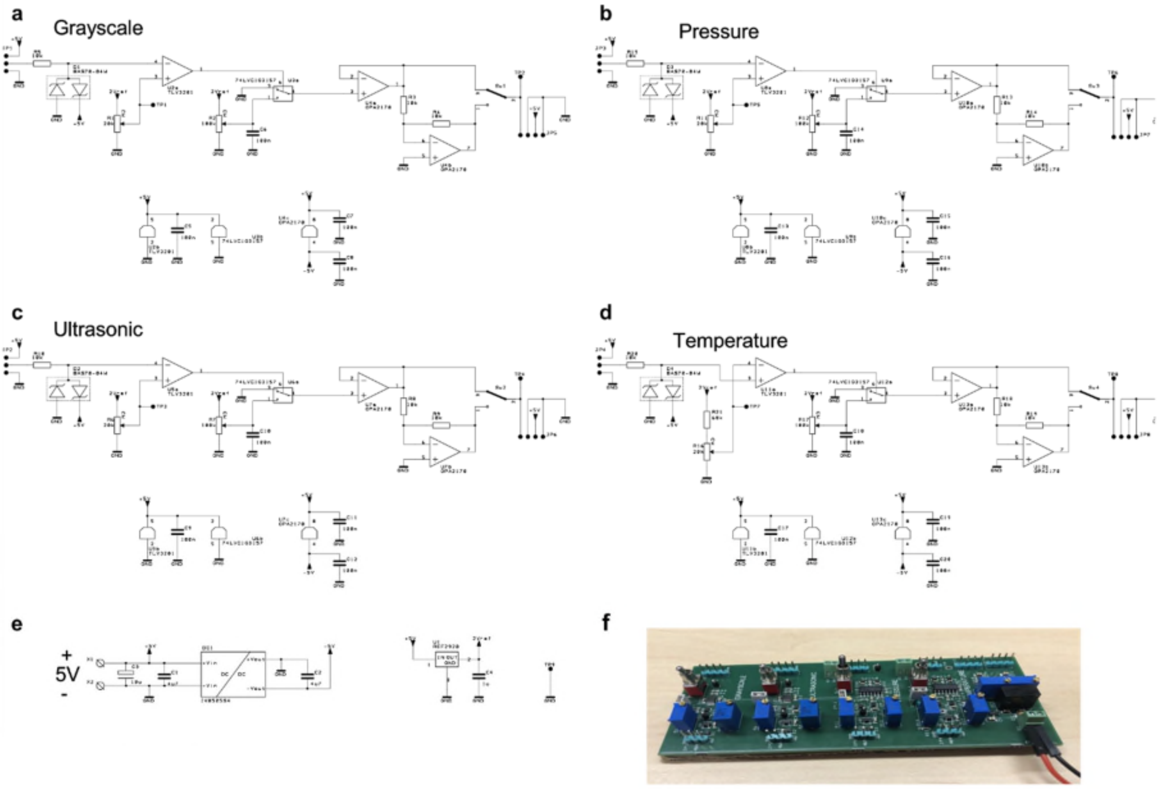


**Fig. S6. Additional hardware circuitry to condition sensor signals.** Circuit diagram of the additional hardware circuitry used to condition the sensor signals. Each sensor signal (grayscale, ultrasonic, pressure and temperature) can be defined in signal strength (voltage) and polarity (-/+) and a threshold can be determined below/above each sensor outputs no signal (0 V). (**a**) For color sensor. (**b**) For pressure sensor. (**c**) For proximity sensor. (**d**) For temperature sensor. (**e**) Circuit to provide different levels and polarities of supply voltage via the Arduino Uno 5V analog output. (**f**) Photograph of the printed circuit board without connected signals and cables.

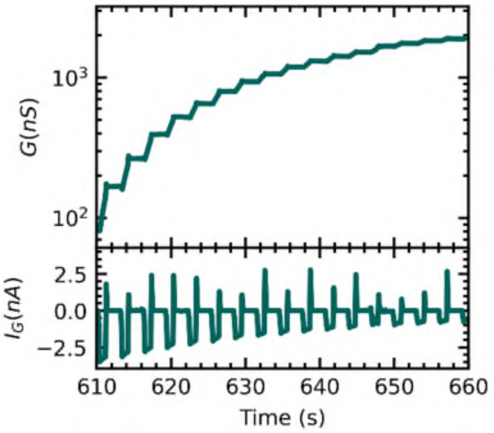


**Figure S7.** Low write currents <5nA of the ECRAM combined with a low transconductance <100nS are promising in terms of energy consumption. A system with similar characteristics (read current ≤10nA for read voltage of 100mV, channel conductance < 100nS) based on organic polymer PEDOT:PSS shows an energy advantage of their system scales with a factor of 476 compared to a conventional static random-access memory (SRAM) architecture[30].

## References


1. Yang, G.-Z., Bellingham, J., Dupont, P. E., Fischer, P., Floridi, L., Full, R., Jacobstein, N., Kumar, V., McNutt, M., Merrifield, R., Nelson, B. J., Scassellati, B., Taddeo, M., Taylor, R., Veloso, M., Wang, Z. L. & Wood, R. The grand challenges of Science Robotics. *Sci. Robot.* **3,** eaar7650 (2018).

2. Hopfield, J. J. Artificial neural networks. *IEEE Circuits Devices Mag.* **4,** 3–10 (1988).

3. Neftci, E. O. & Averbeck, B. B. Reinforcement learning in artificial and biological systems. *Nat. Mach. Intell.* **1,** 133–143 (2019).

4. Bartolozzi, C., Indiveri, G. & Donati, E. Embodied neuromorphic intelligence. *Nat. Commun.* **13,** 1024 (2022).

5. Sandamirskaya, Y., Kaboli, M., Conradt, J. & Celikel, T. Neuromorphic computing hardware and neural architectures for robotics. *Sci. Robot.* **7,** eabl8419 (2022).

6. LeCun, Y., Bengio, Y. & Hinton, G. Deep learning. *Nature* **521,** 436–444 (2015).

7. Krogh, A. What are artificial neural networks? *Nat. Biotechnol.* **26,** 195–197 (2008).

8. Liu, W., Wang, Z., Liu, X., Zeng, N., Liu, Y. & Alsaadi, F. E. A survey of deep neural network architectures and their applications. *Neurocomputing* **234,** 11–26 (2017).

9. Lillicrap, T. P., Santoro, A., Marris, L., Akerman, C. J. & Hinton, G. Backpropagation and the brain. *Nat. Rev. Neurosci.* **21,** 335–346 (2020).

10. Mehonic, A. & Kenyon, A. J. Brain-inspired computing needs a master plan. *Nature* **604,** 255–260 (2022).

11. Zhang, W., Gao, B., Tang, J., Yao, P., Yu, S., Chang, M.-F., Yoo, H.-J., Qian, H. & Wu, H. Neuro-inspired computing chips. *Nat. Electron.* **3,** 371–382 (2020).

12. Furber, S. B., Galluppi, F., Temple, S. & Plana, L. A. The SpiNNaker Project. *Proc. IEEE* **102,** 652–665 (2014).

13. Davies, M., Srinivasa, N., Lin, T.-H., Chinya, G., Cao, Y., Choday, S. H., Dimou, G., Joshi, P., Imam, N., Jain, S., Liao, Y., Lin, C.-K., Lines, A., Liu, R., Mathaikutty, D., McCoy, S., Paul, A., Tse, J., Venkataramanan, G., Weng, Y.-H., Wild, A., Yang, Y. & Wang, H. Loihi: A Neuromorphic Manycore Processor with On-Chip Learning. *IEEE Micro* **38,** 82–99 (2018).

14. Bielecki, J., Nielsen, S. K. D., Nachman, G. & Garm, A. Associative learning in the box jellyfish Tripedalia cystophora. *Curr. Biol.* **33,** 4150-4159.e5 (2023).

15. Howard, D., Eiben, A. E., Kennedy, D. F., Mouret, J.-B., Valencia, P. & Winkler, D. Evolving embodied intelligence from materials to machines. *Nat. Mach. Intell.* **1,** 12–19 (2019).

16. Smith, L. & Gasser, M. The Development of Embodied Cognition: Six Lessons from Babies. *Artif. Life* **11,** 13–29 (2005).

17. Pfeifer, R., Lungarella, M. & Iida, F. Self-Organization, Embodiment, and Biologically Inspired Robotics. *Science* **318,** 1088–1093 (2007).

18. Winding, M., Pedigo, B. D., Barnes, C. L., Patsolic, H. G., Park, Y., Kazimiers, T., Fushiki, A., Andrade, I. V., Khandelwal, A., Valdes-Aleman, J., Li, F., Randel, N., Barsotti, E., Correia, A., Fetter, R. D., Hartenstein, V., Priebe, C. E., Vogelstein, J. T., Cardona, A. & Zlatic, M. The connectome of an insect brain. *Science* (2023). doi:10.1126/science.add9330

19. Skinner, B. F. Selection by Consequences. *Science* **213,** 501–504 (1981).

20. Talin, A. A., Li, Y., Robinson, D. A., Fuller, E. J. & Kumar, S. ECRAM Materials, Devices, Circuits and Architectures: A Perspective. *Adv. Mater.* 2204771 (2022). doi:10.1002/adma.202204771

21. Van De Burgt, Y., Melianas, A., Keene, S. T., Malliaras, G. & Salleo, A. Organic electronics for neuromorphic computing. *Nat. Electron.* **1,** 386–397 (2018).

22. Krauhausen, I., Coen, C.-T., Spolaor, S., Gkoupidenis, P. & van de Burgt, Y. Brain-Inspired Organic Electronics: Merging Neuromorphic Computing and Bioelectronics Using Conductive Polymers. *Adv. Funct. Mater.* **n/a,** 2307729 (2023).

23. Gkoupidenis, P., Zhang, Y., Kleemann, H., Ling, H., Santoro, F., Fabiano, S., Salleo, A. & van de Burgt, Y. Organic mixed conductors for bioinspired electronics. *Nat. Rev. Mater.* 1–16 (2023). doi:10.1038/s41578-023-00622-5

24. Gkoupidenis, P., Schaefer, N., Strakosas, X., Fairfield, J. A. & Malliaras, G. G. Synaptic plasticity functions in an organic electrochemical transistor. *Appl. Phys. Lett.* **107,** 263302 (2015).

25. Van De Burgt, Y., Lubberman, E., Fuller, E. J., Keene, S. T., Faria, G. C., Agarwal, S., Marinella, M. J., Alec Talin, A. & Salleo, A. A non-volatile organic electrochemical device as a low-voltage artificial synapse for neuromorphic computing. *Nat. Mater.* **16,** 414–418 (2017).

26. Kim, Y., Chortos, A., Xu, W., Liu, Y., Oh, J. Y., Son, D., Kang, J., Foudeh, A. M., Zhu, C., Lee, Y., Niu, S., Liu, J., Pfattner, R., Bao, Z. & Lee, T. W. A bioinspired flexible organic artificial afferent nerve. *Science* **360,** 998–1003 (2018).

27. Gkoupidenis, P., Koutsouras, D. A. & Malliaras, G. G. Neuromorphic device architectures with global connectivity through electrolyte gating. *Nat. Commun.* **8,** 15448 (2017).

28. Cucchi, M., Gruener, C., Petrauskas, L., Steiner, P., Tseng, H., Fischer, A., Penkovsky, B., Matthus, C., Birkholz, P., Kleemann, H. & Leo, K. Reservoir computing with biocompatible organic electrochemical networks for brain-inspired biosignal classification. *Sci. Adv.* **7,** eabh0693 (2021).

29. Felder, D., Muche, K., Linkhorst, J. & Wessling, M. Reminding forgetful organic neuromorphic device networks. *Neuromorphic Comput. Eng.* **2,** 044014 (2022).

30. Fuller, E. J., Keene, S. T., Melianas, A., Wang, Z., Agarwal, S., Li, Y., Tuchman, Y., James, C. D., Marinella, M. J., Yang, J. J., Salleo, A. & Talin, A. A. Parallel programming of an ionic floating-gate memory array for scalable neuromorphic computing. *Science* **364,** 570–574 (2019).

31. Liu, F., Deswal, S., Christou, A., Sandamirskaya, Y., Kaboli, M. & Dahiya, R. Neuro-inspired electronic skin for robots. *Sci. Robot.* **7,** eabl7344 (2022).

32. Dai, S., Dai, Y., Zhao, Z., Xia, F., Li, Y., Liu, Y., Cheng, P., Strzalka, J., Li, S., Li, N., Su, Q., Wai, S., Liu, W., Zhang, C., Zhao, R., Yang, J. J., Stevens, R., Xu, J., Huang, J. & Wang, S. Intrinsically stretchable neuromorphic devices for on-body processing of health data with artificial intelligence. *Matter* **5,** (2022).

33. van Doremaele, E. R. W., Ji, X., Rivnay, J. & van de Burgt, Y. A retrainable neuromorphic biosensor for on-chip learning and classification. *Nat. Electron.* **6,** 765–770 (2023).

34. Krauhausen, I., Koutsouras, D. A., Melianas, A., Keene, S. T., Lieberth, K., Ledanseur, H., Sheelamanthula, R., Giovannitti, A., Torricelli, F., Mcculloch, I., Blom, P. W. M., Salleo, A., Burgt, Y. van de & Gkoupidenis, P. Organic neuromorphic electronics for sensorimotor integration and learning in robotics. *Sci. Adv.* **7,** (2021).

35. Harikesh, P. C., Yang, C.-Y., Tu, D., Gerasimov, J. Y., Dar, A. M., Armada-Moreira, A., Massetti, M., Kroon, R., Bliman, D., Olsson, R., Stavrinidou, E., Berggren, M. & Fabiano, S. Organic electrochemical neurons and synapses with ion mediated spiking. *Nat. Commun.* **13,** 901 (2022).

36. Sarkar, T., Lieberth, K., Pavlou, A., Frank, T., Mailaender, V., McCulloch, I., Blom, P. W. M., Torricelli, F. & Gkoupidenis, P. An organic artificial spiking neuron for in situ neuromorphic sensing and biointerfacing. *Nat. Electron.* **5,** 774–783 (2022).

37. Cheng, G., Ehrlich, S. K., Lebedev, M. & Nicolelis, M. A. L. Neuroengineering challenges of fusing robotics and neuroscience. *Sci. Robot.* **5,** eabd1911 (2020).

38. Seminara, L., Dosen, S., Mastrogiovanni, F., Bianchi, M., Watt, S., Beckerle, P., Nanayakkara, T., Drewing, K., Moscatelli, A., Klatzky, R. L. & Loeb, G. E. A hierarchical sensorimotor control framework for human-in-the-loop robotic hands. *Sci. Robot.* **8,** eadd5434 (2023).

39. Iberite, F., Muheim, J., Akouissi, O., Gallo, S., Rognini, G., Morosato, F., Clerc, A., Kalff, M., Gruppioni, E., Micera, S. & Shokur, S. Restoration of natural thermal sensation in upper-limb amputees. *Science* **380,** 731–735 (2023).

40. Honegger, K. & De Bivort, B. Stochasticity, individuality and behavior. *Curr. Biol.* **28,** R8–R12 (2018).

41. Rivnay, J., Inal, S., Salleo, A., Owens, R. M., Berggren, M. & Malliaras, G. G. Organic electrochemical transistors. *Nat. Rev. Mater.* **3,** 1–14 (2018).

42. Melianas, A., Quill, T. J., LeCroy, G., Tuchman, Y., Loo, H. v., Keene, S. T., Giovannitti, A., Lee, H. R., Maria, I. P., McCulloch, I. & Salleo, A. Temperature-resilient solid-state organic artificial synapses for neuromorphic computing. *Sci. Adv.* **6,** eabb2958 (2020).

43. Giovannitti, A., Sbircea, D. T., Inal, S., Nielsen, C. B., Bandiello, E., Hanifi, D. A., Sessolo, M., Malliaras, G. G., McCulloch, I. & Rivnay, J. Controlling the mode of operation of organic transistors through side-chain engineering. *Proc. Natl. Acad. Sci. U. S. A.* **113,** 12017–12022 (2016).

44. Bernards, D. A. & Malliaras, G. G. Steady-state and transient behavior of organic electrochemical transistors. *Adv. Funct. Mater.* **17,** 3538–3544 (2007).

45. Torelli, J. N. & Pickren, S. E. Using Chained or Tandem Schedules With Functional Communication Training: A Systematic Review. *Behav. Modif.* **47,** 185–218 (2023).

46. Kora, P., Ooi, C. P., Faust, O., Raghavendra, U., Gudigar, A., Chan, W. Y., Meenakshi, K., Swaraja, K., Plawiak, P. & Rajendra Acharya, U. Transfer learning techniques for medical image analysis: A review. *Biocybern. Biomed. Eng.* **42,** 79–107 (2022).

47. Köfferlein, M. KLayout - chip mask layout viewing, editing and more. at <https://www.klayout.de/>

48. Coen, C.-T., Krauhausen, I. & Spolaor, S. koala: KlayOut mAsk Layout Automation. at <https://pypi.org/project/koala/>

49. Lee, K. H., Kang, M. S., Zhang, S., Gu, Y., Lodge, T. P. & Frisbie, C. D. ‘Cut and stick’ rubbery ion gels as high capacitance gate dielectrics. *Adv. Mater.* **24,** 4457–4462 (2012).
